# Supplementary material for: Yiai Fuzheng decoction inhibits triple-negative breast cancer by remodeling the immune microenvironment
Source: Front Immunol. 2025 Sep 30;16:1615631. doi: 10.3389/fimmu.2025.1615631 (PMC12518410; doi:10.3389/fimmu.2025.1615631)
Supplement: Supplementary file 2 [file DataSheet1.zip › Supplementary File 2/Treatment/C_vs_D/07_Pathway_Analysis/Pathway_Analysis/Sig_Pathways_Name/Glyoxylate and dicarboxylate metabolism.pdf]

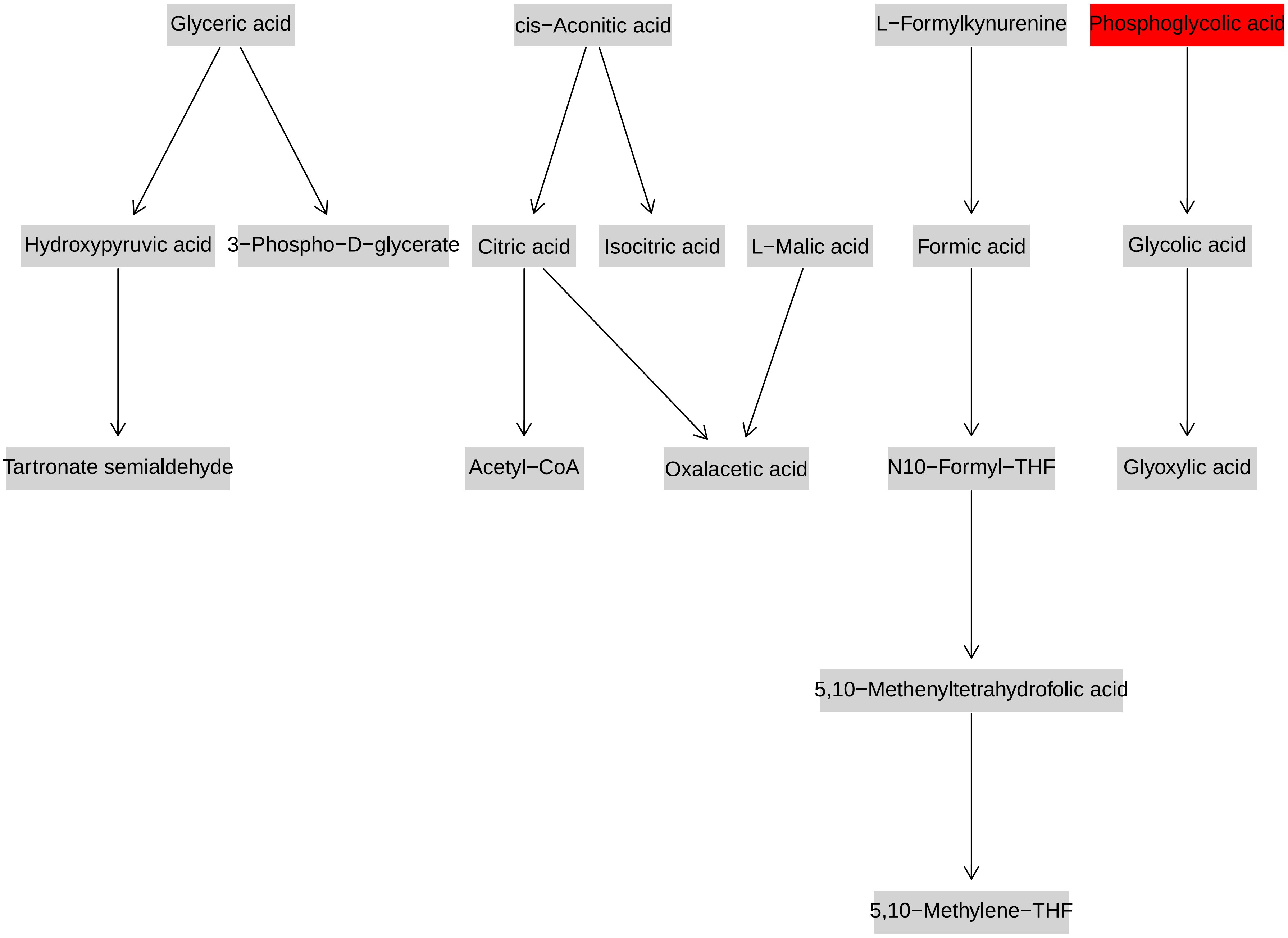

Glyceric acid
cis−Aconitic acid
L−Formylkynurenine
Phosphoglycolic acid
Hydroxypyruvic acid
3−Phospho−D−glycerate
Citric acid
Isocitric acid
L−Malic acid
Formic acid
Glycolic acid
Tartronate semialdehyde
Acetyl−CoA
Oxalacetic acid
N10−Formyl−THF
Glyoxylic acid
5,10−Methenyltetrahydrofolic acid
5,10−Methylene−THF
